# Synergetic Effect of Graphene and MWCNTs on Microstructure and Mechanical Properties of Cu/Ti_3_SiC_2_/C Nanocomposites

**DOI:** 10.1186/s11671-017-2378-0

**Published:** 2017-11-28

**Authors:** Xiaosong Jiang, Tingfeng Song, Zhenyi Shao, Wanxia Liu, Degui Zhu, Minhao Zhu

**Affiliations:** School of Materials Science and Engineering, Southwest Jiaotong University, Chengdu, Sichuan 610031 China

**Keywords:** Multi-walled carbon nanotubes, Graphene, Cu/Ti_3_SiC_2_/C Nanocomposites, Hot isostatic pressing, Microstructure

## Abstract

Multi-walled carbon nanotubes (MWCNTs) and graphenes have been taken for novel reinforcements due to their unique structure and performance. However, MWCNTs or graphenes reinforced copper matrix composites could not catch up with ideal value due to reinforcement dispersion in metal matrix, wettability to metal matrix, and composite material interface. Taking advantage of the superior properties of one-dimensional MWCNTs and two-dimensional graphenes, complementary performance and structure are constructed to create a high contact area between MWCNTs and graphenes to the Cu matrix. Mechanical alloying, hot pressing, and hot isostatic pressing techniques are used to fabricate Cu matrix self-lubricating nanocomposites. Effects of MWCNTs and graphenes on mechanical properties and microstructures of Cu/Ti_3_SiC_2_/C nanocomposites are studied. The fracture and strengthening mechanisms of Cu/Ti_3_SiC_2_/C nanocomposites are explored on the basis of structure and composition of Cu/Ti_3_SiC_2_/C nanocomposites with formation and function of interface.

## Background

Copper-graphite composite has good electrical conductivity, high thermal conductivity, good wear resistance, and other properties; in that, it is a blend of advantages of copper and graphite, so it is increasingly used in aerospace, electronics, automotive application, and other fields [1, 2]. Ti_3_SiC_2_ offers advantages of heat conductivity, high electric conduction, easy processing similar to metals, oxidation resistance, light weight, and high temperature resistance, making it a useful material for multiple applications [3, 4]. Without affecting the self-lubricating properties and conductivity of copper graphite alloys, Ti_3_SiC_2_ can improve the strength, hardness, and wear resistance of copper-graphite composites [5]. Cu/Ti_3_SiC_2_/C composites are a new promising material system that combines advantages of copper-graphite composites and Ti_3_SiC_2_, but the mechanical properties, wear resistance, and other performance parameters of these materials remain insufficient under certain conditions [6].

The introduction of uniformly dispersed nanoscale reinforcement particles into a metal matrix results in metal matrix composites that may have better mechanical properties, electrical conductivity, thermal conductivity, wear resistance, corrosion resistance, and high temperature and oxidation resistance [7]. One-dimensional multi-walled carbon nanotubes (MWCNTs) and two-dimensional graphene are attractive materials for composite reinforcement due to their unique structure and performance [8–10] and are being used in place of graphite to prepare metal matrix composites [11–13]. Nevertheless, the performances of copper matrix composites made from MWCNTs or graphene remain insufficient. There are many factors that limit the performance of these materials: reinforcement dispersion in metal matrix, wettability to metal matrix, and the metal matrix interface.

The potential for integration of two-dimensional materials into new hetero-structures bound by weak van der Waals interactions was demonstrated by a forward-looking analysis, examining the possibility of combining graphene with other structures [14, 15], and the feasibility of this approach has been demonstrated [16]. Most studies on synergistic enhancements by MWCNTs and graphene have focused on polymer matrix composites in which a continuous interconnected network can be achieved by synergistic reinforced mechanism [17–19]. However, there are still some difficulties in the enhancement of metal matrix composites by MWCNTs and graphene. There are challenges in the use of one-dimensional MWCNTs and two-dimensional graphene to achieve the properties of three-dimensional braided composite material, which can produce synergistic cooperative and multi-scale reinforcements [20–22]. In this work, Cu/Ti_3_SiC_2_/C composites with both MWCNTs and graphene were prepared by mechanical alloying, vacuum hot-pressing (VHP), and hot isostatic pressing (HIP) techniques. Surface modifications of MWCNTs and graphene were conducted to improve their dispersion. Microstructure and mechanical properties of the prepared Cu/Ti_3_SiC_2_/C composites were measured to evaluate the reinforcing effects. Based on the experimental results, the strengthening and fracture mechanisms of Cu/Ti_3_SiC_2_/C composites are discussed.

## Methods/Experimental

Taking advantage of the superior properties of one-dimensional carbon nanotubes and two-dimensional graphene, synergistically strengthened nano-structure was designed to prepare Cu/Ti_3_SiC_2_/C nano-composites by a multi-phase synergistically strengthening process. Mechanical alloying, hot pressing, and HIP techniques were used to fabricate Cu/Ti_3_SiC_2_/C nanocomposites under both elevated temperature and high pressure. The properties of the raw material powders of MWCNTs, graphene nanoplatelets (GNPs), electrolytic copper powder graphite powder, and Ti_3_SiC_2_ powder used in this experiment are listed in Table 1. Dispersions of MWCNTs and graphene were assisted by ultrasonic oscillation; plasma and chemical treatment surface modification were performed using Ar-NH_3_ plasma and 0.02μg/ml Rutin or 10 μg/ml Gallic acid solution [23, 24]. Composition design details of the nanocomposites are listed in Table 2. Materials were mixed by high-energy ball milling with an agate milling ball, and the nanocomposite powder was processed at a10:1 mass ratio using tert butyl alcohol as the dispersing medium. The mixed powders were sintered according to the setting process (vacuum hot-pressing: 950 °C × 20 MPa × 2 h, hot isostatic pressing: 900 °C × 100 MPa × 2 h) to obtain the Cu-matrix nanocomposite [25].The relative densities of nanocomposite materials were analyzed by density measurement with liquid drainage based on Archimedes law (Table 3). Microstructures of Cu/Ti_3_SiC_2_/C nanocomposites were characterized by optical microscopy (OM, AxioCam MRC5), X-ray diffraction (XRD, X’Pert Pro-MPD) analysis, scanning electron microscope (SEM, JEOL JSM-7001F at 15 kV) with an energy dispersive X-ray spectrometer (EDS), and transmission electron microscope (TEM, FEI Tecnai F20ST at 200 kV). The hardness measurement was determined by using a Vickers hardness (HV, HXD-1000TM) tester. Tensile, compression, and shearing tests of Cu/Ti_3_SiC_2_/C nanocomposites were performed using a microcomputer-controlled electronic universal testing machine (WDW-3100) at a loading speed of 0.5 mm/min. Effects of MWCNTs and GNPs on the properties and microstructures of the prepared Cu matrix nanocomposites were determined.

**Table 1:** Properties of raw material powders

Material	Density(g/cm^3^)	Size	Purity (%)
MWCNTs	2.1	Diameter 20~30 nm, length 10~30 μm	≥ 95
Graphenes	2.1	≤ 5 layers	≥ 99.8
Ti_3_SiC_2_	4.53	200 mesh	
C	2.2	250 mesh	
Cu	8.89	300 mesh	≥ 99.99

**Table 2:** Composition design details of the nanocomposites (wt%)

Graphenes	MWCNTs	Cu	Ti_3_SiC_2_	C	La
0.2	0.8	85.9	10	3	0.1
0.5	0.5	85.9	10	3	0.1
0.8	0.2	85.9	10	3	0.1

**Table 3:** Relative densities of nanocomposite materials

Graphenes/MWCNTs (wt%)	Theory density (g/cm^3^)	Actual density (g/cm^3^)	Relative density (%)
0.2:0.8	7.21	7.29	98.90
0.5:0.5	7.24	7.29	99.31
0.8:0.2	7.03	7.29	96.43

## Results and Discussion

### Powder Microstructure and Phase Identification

**Fig. 1:**
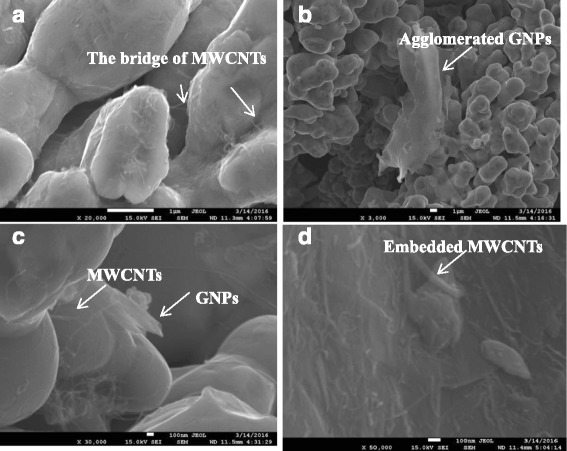
SEM images of raw materials of Cu, Ti_3_SiC_2_, C, MWCNT, and graphene powders after ball milling, taken at a low magnification (**b**) and a high magnification (**a**, **c**, **d**). **a**–**c** Nanocomposites with 0.5 wt% graphenes and 0.5 wt% MWCNTs. **d** Nanocomposites with 0.8 wt% graphenes and 0.2 wt% MWCNTs

Mechanical alloying is a technique that involves a series of physical and chemical processes such as repeated deformation, cold-welding, and fracturing for the composite powders using high-energy ball milling. The powders are ground to micron-size or even nano-size. They are well mixed to produce composite powder. This milling is expected to improve the homogeneous dispersion of GNPs/MWCNTs in the copper matrix by mechanical alloying and achieve desirable interface bonding by miniature forging, thus improving the mechanical properties of the resulting materials. The SEM images of Cu, Ti_3_SiC_2_, C, MWCNT, and GNP powders after ball milling are shown in Fig. 1. The larger particles are Cu, and the smaller particles that are uniformly dispersed between the Cu particles areTi_3_SiC_2_ and C. At a higher magnification, the bridging state of MWCNTs and embedded state of GNP powders, as indicated by arrows in Fig. 1, were dispersed on the peripheral surfaces of Cu particles. Cold welding enables the copper matrix to be well bounded to GNPs/MWCNTs. GNPs with certain transparency and winding linear MWCNTs were distributed on the surface or in the interior of the copper particle agglomerate. As shown in Fig. 1a, most MWCNTs are disordered and distributed on the surface of the copper particles. MWCNTs formed a bridge between copper particle agglomerates as shown in the inlay and indicated by arrows. This indicates that the ball-milling process promotes interface bonding between the reinforcement MWCNTs and the matrix [26]. As shown in Fig. 1b, agglomerate GNPs were distributed on the surface of the copper matrix. Agglomeration occurs in GNPs, as shown by the arrows. The intrinsic extremely large specific surface area of GNPs and the presence of van der Waals force increased the susceptibility to agglomeration, decreased the dispersion uniformity, and reduced the interface bonding. In Fig. 1c, as shown by the arrows, small size GNPs are inlaid into the matrix due to the mechanical action of ball milling, collision, and friction between composite powder particles and GNPs during ball milling, thus improving the interface bonding strength. Nevertheless, many wrinkles occur in GNPs, thus reducing the effective contact area between GNPs and the matrix. In Fig. 1d, as indicated by the arrows, MWCNTs are inlaid into the Cu matrix and distributed on the surface of Cu particles in a disordered manner. In general, the ball-milling mixing process is efficient, resulting in the effective inlay of some GNPs/MWCNTs into the copper matrix particles. Nevertheless, the agglomeration of GNPs/MWCNTs is still very severe.

**Fig. 2:**
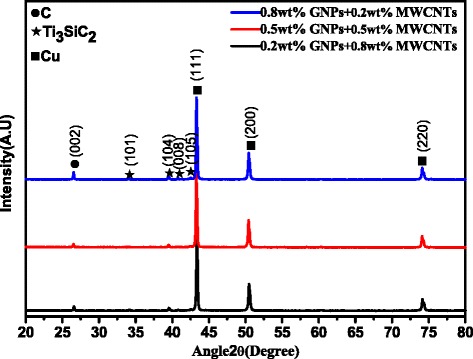
XRD patterns of raw materials after ball milling. Nanocomposites with 0.8 wt% graphenes and 0.2 wt% MWCNTs, nanocomposites with 0.5 wt% graphenes and 0.5 wt% MWCNTs, and nanocomposites with 0.2 wt% graphenes and 0.8 wt% MWCNTs

The XRD results of raw materials including Cu, Ti_3_SiC_2_, C, MWCNTs, and GNPs after ball milling are shown in Fig. 2. The results show changes in the new phase identification from raw materials to the mechanical alloying process. Cu, Ti_3_SiC_2_, and graphite were detected as shown in Fig. 2, indicating that phase identification did not occur during the mechanical alloying process. No diffraction peaks were observed for CuO or Cu_2_O, indicating that the copper powder was not oxidized, the decomposition reaction did not occur for Ti_3_SiC_2_, and no chemical reaction occurred.

### Nanocomposite Phase and Microstructure Identification

The compactness of nanocomposites prepared with 0.2 wt% graphene and 0.8 wt% MWCNTs approximates the compactness of nanocomposites prepared with 0.5 wt% graphene and 0.5 wt% MWCNTs. However, the compactness decreased to 96.43% when the composition was 0.8 wt% graphene and 0.2 wt% MWCNTs. As mentioned above, with the increase of the content of GNPs, the agglomeration of reinforcement phase exhibits an increasing trend and thus weakens its effect to refine grains and impedes sintering and bonding, formation and growth of the sintering neck, and gap closing between Cu particles. In this way, the GNPs can influence diffusion and migration between Cu atoms and increase porosity. Therefore, the compactness of sintered nanocomposites decreased with increased GNPs. In the experiment, the compactness of nanocomposites prepared with 0.2 wt% graphene and 0.8 wt% MWCNTs was less than that of nanocomposites prepared with 0.5 wt% graphene and 0.5 wt% MWCNTs, but this difference is very slight. Consequently, GNP/MWCNT synergistic enhancement increased reinforcement agglomeration and decreased the grain refinement effect, hindering Cu particles sintering, the formation and growth of sintered necks, and the gap closure process. Overall, the GNPs/MWCNTs affected the diffusion of Cu atoms between the matrix and reinforcements to reduce interfacial bonding and increase nanocomposite porosity.

**Fig. 3:**
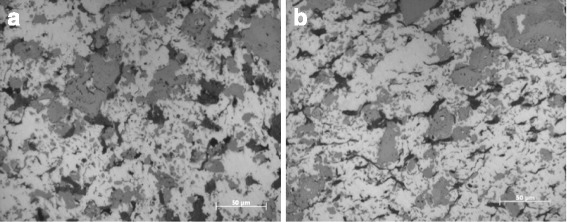
Metallographic results of sintered nanocomposites with 0.8 wt% graphenes and 0.2 wt% MWCNTs in different directions (**a** is parallel to the pressure and **b** is a perpendicular to the pressure)

In the metallographic microstructure information presented in Fig. 3, the white structure is the Cu matrix, the gray structure is Ti_3_SiC_2_, and the black part is C or the hole. The Cu phases are basically connected to form the matrix, which is a discontinuous network-like structure distributed on Ti_3_SiC_2_ or TiC. But the graphite is distributed in a completely isolated manner so that most of the graphite is uniformly distributed in the Cu matrix in a smallish, worm-like shape or an irregular flocculent shape, thus improving the antifriction lubricating property of the sintered nanocomposite. During sintering of the nanocomposites, Cu particles do not interact with the Ti_3_SiC_2_ and the graphite powder. The semi-melted Cu particles are sintered into the copper matrix after contacting each other to form a bonding surface that allows the formation and growth of a sintering neck and the formation of closed pores. The original mechanical engagement transforms into interatomic metallurgical bonding. The graphite or agglomerated GNPs and MWCNTs are retained in closed pores to form the black area evident in the metallographic microstructure. The Ti_3_SiC_2_ powder is also subjected to a sintering process with Cu particles, thus forming the gray Ti_3_SiC_2_ region in the metallographic microstructure. According to the researching report on XRD analysis of the similar sintered nanocomposites, the differences are as follows: Ti_3_SiC_2_ is decomposed into Si and TiC at a sintering temperature of 950 °C [27]. Si atoms and the copper matrix react to produce Cu_9_Si, which increases the interface bonding between the Cu matrix and Ti_3_SiC_2_ [27].

The metallurgical phase of nanocomposites is shown in Fig. 3 in the longitudinal and traverse directions relative to the hot pressing direction. There are significant differences in the metallurgical phase microstructure perpendicular and parallel to the hot pressing direction. The graphite in the black region perpendicular to the hot-pressing direction exhibits a flocculent shape, and the graphite in the black region parallel to the hot-pressing direction exhibits a more slender shape. The reasons for this difference are as follows: The hot pressing sintering process is single-action pressing. Vertical pressure is applied on the composite powder in a cylindrical graphite die. There are significant morphological differences between the graphite perpendicular to the hot-pressing direction and the graphite parallel to the hot-pressing direction, because the graphite is intrinsically fluffy. These differences are maintained after isostatic pressing, because the pressures applied to the composite green body are equal in every direction during isostatic pressing after the hot pressing sintering step. Thus, the morphological differences formed during hot pressing sintering remain.

**Fig. 4:**
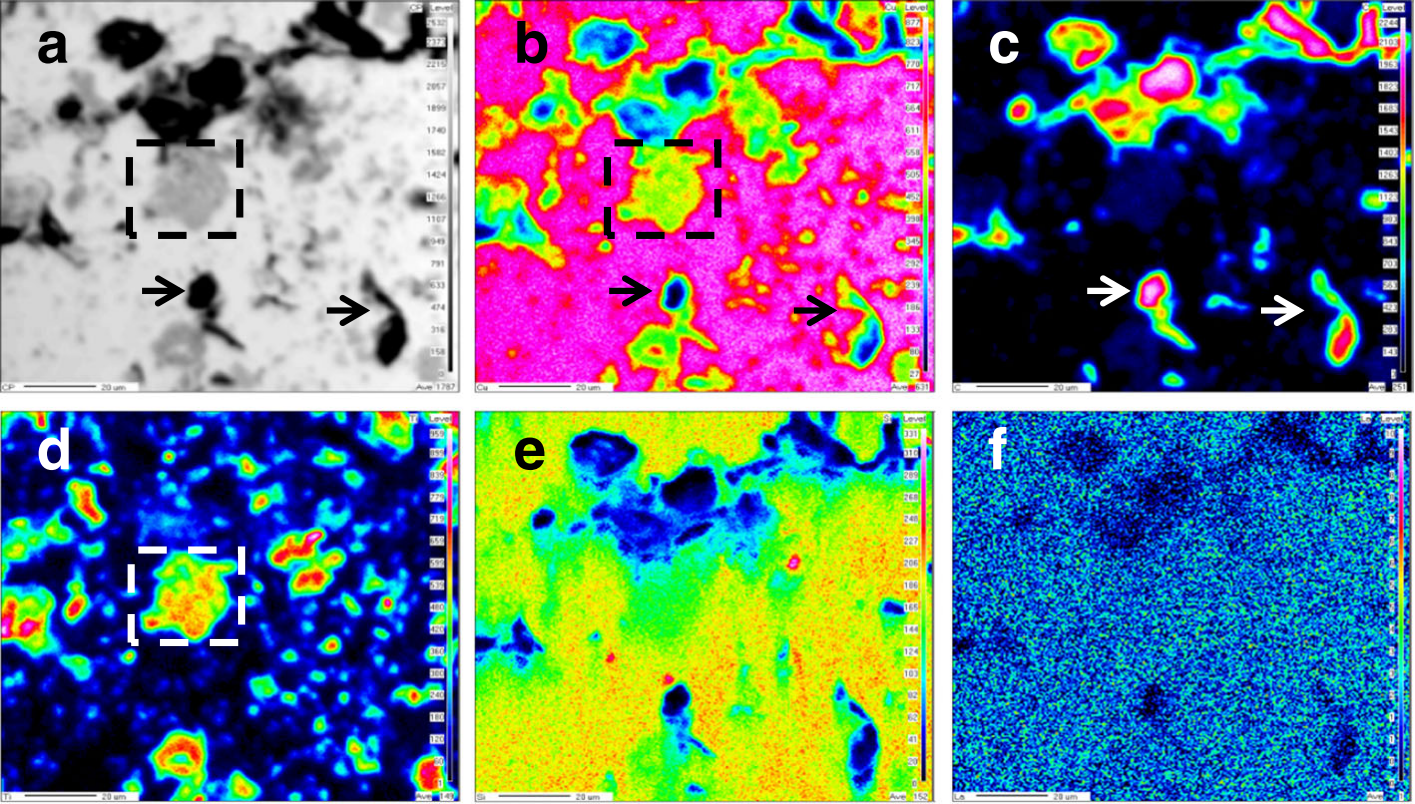
EPMA analyses of sintered nanocomposites with 0.2 wt% graphenes and 0.8 wt% MWCNTs. **a** BS image, **b** Cu map, **c** C map, **d** Ti map, **e** Si map, and **f** La map

To further identify the products, the nanocomposite microstructure was analyzed by EPMA for elemental mapping. As shown in Fig. 4a, the backscattered (BS) image shows Cu grains at a higher contrast, and Ti_3_SiC_2_ and C were located between Cu grains with darker contrast. Based on the distribution of Cu in the sintered nanocomposites shown in Fig. 4b, the red region represents Cu and is the majority of the material. The red bright spots in Fig. 4c represents C element. It can be inferred that agglomeration occurs in the nanocarbon. Based on the observation of the distribution of Ti in Fig. 4d, the yellow irregular bright spot distributed throughout the whole matrix is Ti, corresponding to the gray phase in Fig. 4a. Similarly, the green bright spots in Fig. 4e represent the distribution of Si in the sintered nanocomposites. Based on this observation, Si is distributed uniformly. La is also dispersed in the sintered nanocomposites, and the red bright spots in Fig. 4f represent La. La is added to increase the compactness of sintered nanocomposites. The observed homogeneous dispersion in the sintered nanocomposites is very important for the resulting properties of the prepared materials.

Light gray spots of Ti_3_SiC_2_were observed and are indicated in the dotted line box in Fig. 4a, and Cu and Ti are present in the dotted line box in Fig. 4b, d. Within the dotted line box in Fig. 4b, the color changes from bright red to green from the matrix to the center of the bright spots, indicating that the concentration of Cu is decreasing gradually. Within the dotted line box in Fig. 4c, the color of Ti changes from orange in interior to green in exterior. It can be inferred that the concentration of Ti_3_SiC_2_ is decreasing gradually. These results suggest that the Cu phase and Ti_3_SiC_2_ phase are closely bound and Cu and Ti_3_SiC_2_ have high wettability [27].

Based on the observation and comparison of the areas indicated by the arrows in Fig. 4a-c, it can be inferred that the black tadpole-like substance primarily comprises C. As shown in Fig. 4c, the center of C is bright red, indicating that the concentration of C is high. A green circle around the bright red spots indicates that the concentration of C decreases gradually from the center of the bright spots to the exterior. As indicated by the arrows in Fig. 4b, the color changes from bright red to green and then the final color is dark blue, moving from the matrix to the center of the bright spots. The changes in color indicate that the concentration of Cu is decreasing gradually. Such gradual change processes of the two elements verify the mutual diffusion of C and Cu, suggesting close binding between the C reinforcement phase and the Cu matrix. The content of Cu in the red dotted line box is high and stable, and the corresponding content of C is low but stable, indicating a small amount of C diffuses in Cu. Cu and C would not react to produce new substances under the conditions of the experiment. These results suggest that C partially diffuses into Cu, and Cu and C form are well bound.

**Fig. 5:**
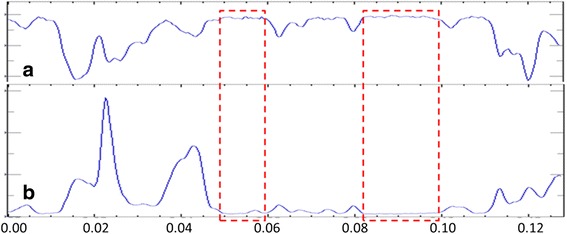
Element distribution line scanning of sintered nanocomposites with 0.2 wt% graphenes and 0.8 wt% MWCNTs. **a** Cu and **b** C

Figure 5 presents the linear scan results at a random position of nanocomposites prepared with 0.2 wt% graphene and 0.8 wt% MWCNTs. Figure 5a shows the line distribution of Cu elements, and Fig. 5b is the line distribution of the C element. The Cu element content is high at the red dotted line box and remains stable. The corresponding C element content is low and also remains stable. This may serve to explain, at least in part, diffusion of a small amount of C into Cu because Cu and C in this experimental condition will not react to generate a new phase. Therefore, Cu and C area good combination as carbon atoms can diffuse in copper atoms.

**Fig. 6:**
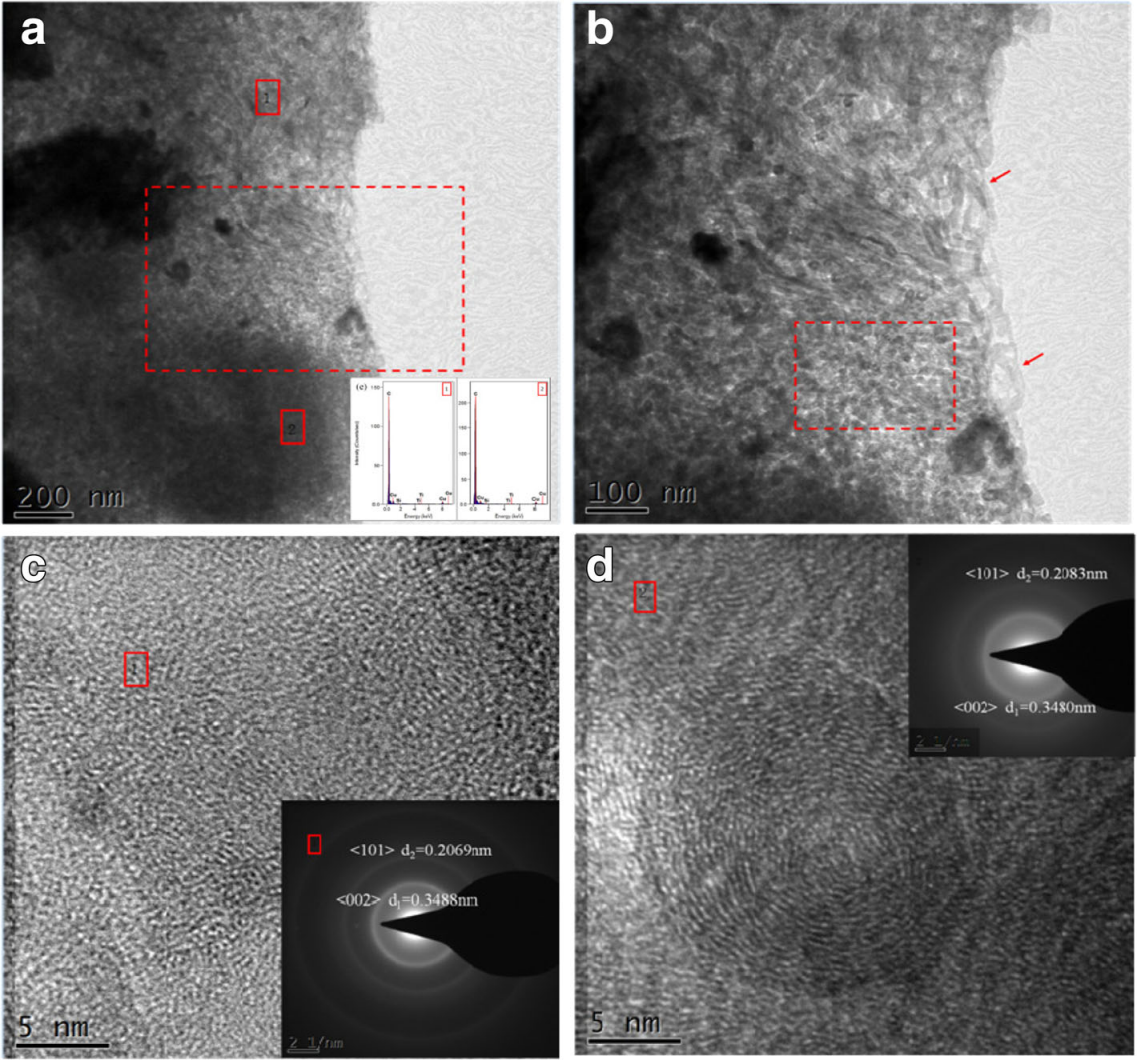
**a**–**d** TEM image in sintered nanocomposites with 0.5 wt% graphenes and 0.5 wt% MWCNTs. **a** TEM image showing nano-Carbon microstructure in Cu; **b** Magnified TEM image of Fig. 6a; **c** High resolution TEM image analysis taken from the marked cross symbol spots 1 in Fig. 6a; **d** High resolution TEM image analysis taken from the marked cross symbol spots 2 in Fig. 6a. Inserted in **a** is EDS spectra taken from the marked cross symbol spots 1and 2

TEM image of the prepared nanocomposites with 0.5 wt% graphene and 0.5 wt% MWCNTs is shown in Fig. 6. Figure 6a is TEM image showing nano-carbon microstructure in Cu matrix, and Fig. 6b is magnified TEM image of Fig. 6a. in which the microstructure of nano-carbon that can be found in that figure, inserted in Fig. 6a, is the EDS spectra taken from the marked cross symbol spots 1and 2. Figure 6c, d is a high-resolution TEM image analysis taken from the marked cross symbol spots 1 and 2 in Fig. 6a. When coupled with the results of morphological and EDS, leads to conclusion that nano-Carbon are solid rod-shaped, its atomic arrangement arrange is significantly different with copper atoms and combine well, while diffraction rings in Fig. 6c, d indicate GNPs and MWCNTs’ agglomeration is serious [28].

**Fig. 7:**
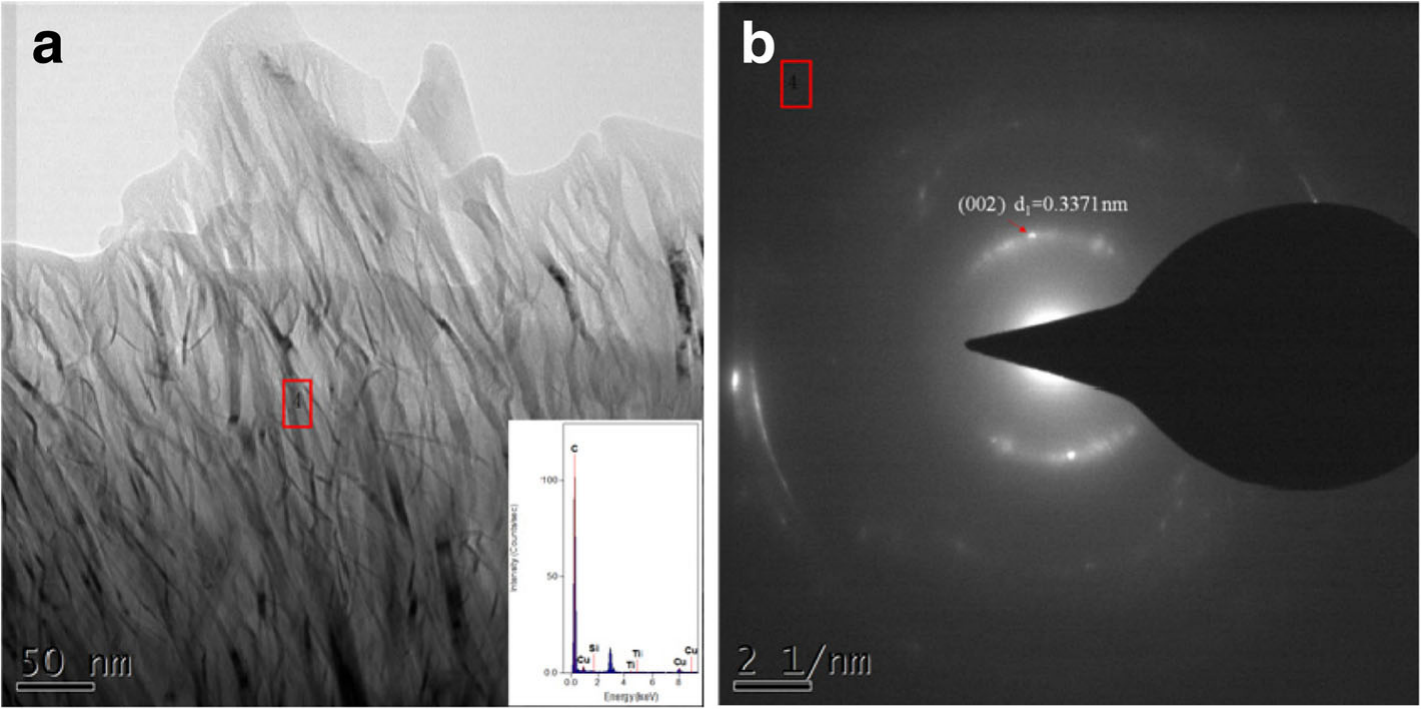
**a** TEM image of graphenes in sintered nanocomposites with 0.5 wt% graphenes and 0.5 wt% MWCNTs. **b** Diffraction patterns from Fig. 7a. Inserted in Fig. 7a is a EDS spectra taken from Fig. 7a

Figure 7a is the TEM image of graphene in the sintered nanocomposites prepared with 0.5 wt% graphene and 0.5wt% MWCNTs. From Fig. 7a, it can be seen that graphene is present in the Cu matrix. The result inserted in Fig. 7a is the EDS spectra taken from Fig. 7a. The graphene appears translucent, the edges are curled, and the graphene is still mostly in a monolayer in the composite, with even distribution.

**Fig. 8:**
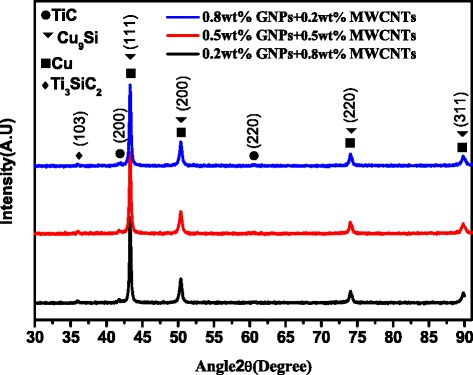
XRD patterns of sintered nanocomposites: Nanocomposites with 0.8 wt% graphenes and 0.2 wt% MWCNTs, nanocomposites with 0.5 wt% graphenes and 0.5 wt% MWCNTs, and nanocomposites with 0.2 wt% graphenes and 0.8 wt% MWCNTs

Figure 8 shows the XRD patterns of the nanocomposites in which TiC and Cu_9_Si were detected. These results are similar to the results in Fig. 2 of the nanocomposite powders and three types of Cu/Ti_3_SiC_2_/C/graphene composites. The Ti_3_SiC_2_ is decomposed at high temperature, and the decomposition products and Cu matrix will react to generate Cu_9_Si. At a high temperature, the special interlayer weakly-bonding structure of Ti_3_SiC_2_ can make it easy for Si atom to break the restraint bonding to form frees Si. The decomposed Si enters the Cu matrix to form Cu-Si solid solution [27]. TiC is a decomposition product of Ti_3_SiC_2_ during sintering as Si atoms separate from Ti_3_SiC_2_ [27]. TiC is a hard brittle phase with a high melting point, so it has high hardness characteristics. Cu_9_Si is also a hard brittle phase. Both TiC and Cu_9_Si can cause stress concentration, which can reduce performance of the nanocomposites.

The thermodynamic analysis of the possible reactions can be analyzed through the following reactions during the sintering process of composites [11]:

1 $$ {\mathrm{Ti}}_3{\mathrm{SiC}}_2=3{\mathrm{Ti}\mathrm{C}}_{2/3}+\mathrm{Si} $$

2 $$ \mathrm{C}+\mathrm{Si}=\mathrm{SiC} $$

3 $$ \mathrm{SiC}+9\mathrm{Cu}={\mathrm{Cu}}_9\mathrm{Si}+\mathrm{C} $$

4 $$ \mathrm{Si}+9\mathrm{Cu}={\mathrm{Cu}}_9\mathrm{Si} $$

The Gibbs free energy of reaction (1) in which Ti_3_SiC_2_ is decomposed can be calculated as: Δ_*r*_ *G*_*m*_ =  ∑ *ν*_*B*_Δ_f_ *G*_m_ =  ‐ 106.52kJ/mol < 0 indicating that the reaction can occur [11]. For reaction (2), the Gibbs free energy of the reaction can be calculated as: (4) − (3) = (2), and the Δ_r_Gm of the reaction (2) is − 66.99 kJ/mol. Thus, reaction (4) tends to occur more frequently than reaction (3), which is consistent with the absence of SiC compounds in the XRD phase analysis.

### Mechanical Properties and Tensile Fracture Analysis of Nanocomposite Materials

Samples were tested using a Micro Vickers Hardness Tester in which the composite hardness is given as

5 $$ \mathrm{HV}=\frac{0.102F}{A}=0.1891\frac{F}{d^2} $$

where HV is the Micro Vickers hardness, *F* is the loading (gf), *A* is the surface area of indentation pits (mm^2^), and *d* is the residual indentation of two diagonal lengths, *d* = (d1 + d2)/2.

The results of the micro Vickers hardness tests for the nanocomposites were determined and are listed in Table 4. The microhardness of the sintered nanocomposites exhibited a slightly decreasing trend, decreasing to 96.859 from 97.787 when the composition changed from 0.5 wt% graphene and 0.5 wt% MWCNTs to 0.2 wt% graphene and 0.8 wt% MWCNTs. However, the microhardness of the sintered nanocomposites decreased significantly by 9.4%, decreasing to 88.626 from 97.787, when the content of GNPs increased to 0.8 wt%. Overall, with the increase of the content of GNPs, the hardness of the sintered nanocomposites exhibited a decreasing trend. Fundamental causes of the trend are as follows: (1) With the increase of the content of GNPs, agglomeration is more likely to occur in GNPs than in MWCNTs, as GNPs have a larger diameter. Finally, agglomeration occurred in the reinforcement phase as GNPs and MWCNTs increased. The agglomeration thus decreases the number of the reinforcement phases that transmit load and decrease the hardness of the sintered nanocomposites [11, 13, 22]. Sintering decreases binding between the matrixes, increases the void fraction, and decreases the compactness and the hardness of the sintered nanocomposites [11, 13, 22]. (2)With the increase of the content of GNPs, the corresponding content of MWCNTs decreases.

**Table 4:** Mechanical properties of nanocomposite materials

Item	HV	Compressive strength (MPa)	Tensile strength (MPa)	Shear strength (MPa)
Cu/Ti_3_SiC_2_/C^①^	97.787 (± 4.6847)	468.94 (± 4.33)	177.23 (± 3.23)	108.38 (± 11.17)
Cu/Ti_3_SiC_2_/C^②^	96.859 (± 2.7743)	459.03 (± 7.26)	176.24 (± 1.85)	104.49 (± 0.98)
Cu/Ti_3_SiC_2_/C^③^	88.626 (± 3.3447)	412.87 (± 1.81)	156.11 (± 4.91)	86 (± 4.96)
Cu/Ti_3_SiC_2_/C[6]	85	/	113	/
Cu/Ti_3_SiC_2_/C/				
MWCNTs [10]	91	301.8	126.92	/

The specimens of Cu/Ti_3_SiC_2_/C ^①^, Cu/Ti_3_SiC_2_/C ^②^, and Cu/Ti_3_SiC_2_/C ^③^ are nanocomposites with 0.8 wt% graphenes and 0.2 wt% MWCNTs, nanocomposites with 0.5 wt% graphenes and 0.5 wt% MWCNTs, and nanocomposites with 0.2 wt% graphenes and 0.8 wt% MWCNTs

The measured mechanical properties of the nanocomposites are listed in Table 4. When the soft coefficient of stress (α) of the uni-axial compression test is 2, it is softer than the tensile stress state and can show mechanical behavior of the brittle material in the plastic state. From Table 4, the compressive strength of the nanocomposites decreased with the increase of GNPs content. The compressive strength differences of the nanocomposites were within 2% for the nanocomposites prepared with 0.2 wt% and 0.5 wt% of GNPs. The material prepared with 0.8 wt% GNPs showed a decrease of 12% in the compressive strength. Similarly, the tensile strength of the nanocomposites declined 12%. For nanocomposites synergistically strengthened by GNPs and MWCNTs, the GNPs and MWCNTs agglomeration increases with increased amount of GNPs, which can lead to the uneven distribution of GNPs and MWCNTs in the nanocomposites. The formation of irregular micro-cracks also increased in the compression sintering process, and the interfacial bonding with the copper matrix was poor due to the agglomeration, eventually leading to reduced compressive strength and tensile strength of the nanocomposites [22].

Table 4 also shows the shear strength data of the nanocomposites. As shown above, the general trend appears to be decreased shear strength as the GNPs content increases. The shear strength differences of the nanocomposites are small (only down to 3.6%) for the nanocomposites with 0.2 wt% and 0.5 wt% of GNPs. However, for the 0.8 wt% GNP material, the shear strength of the nanocomposites declined 20.6%. The observed shear strength change trend of the nanocomposites is consistent with the tensile and compressive strength of the nanocomposites. That is because the shear strength reflects the material cohesion including the atomic or intermolecular interconnection force, so shear strength can be used as an indicator of interface binding strength between the copper matrix and the strengthening phase. GNP and MWCNT agglomeration increased with the increase of GNP contents, which can lead to the uneven distribution of GNPs and MWCNTs in the nanocomposites. Simultaneously, the huge specific surface area and small thickness between graphene and the copper matrix allow interfacial bonding and shear strength transferring [29]. Thereby, a decline in the shear-lagging enhancement phase could result in a decrease of the shear strength of the nanocomposite material.

**Fig. 9:**
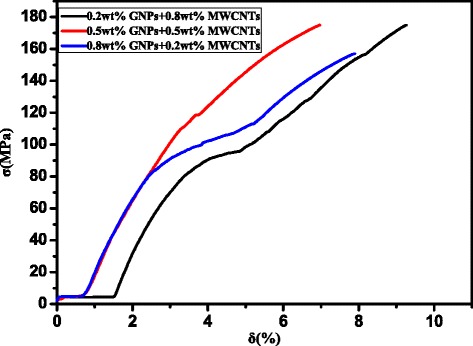
Tensile deformation curves of sintered nanocomposites: Nanocomposites with 0.8 wt% graphenes and 0.2 wt% MWCNTs, nanocomposites with 0.5 wt% graphenes and 0.5 wt% MWCNTs, and nanocomposites with 0.2 wt% graphenes and 0.8 wt% MWCNTs

Tensile deformation curves of nanocomposites were determined and are shown in Fig. 9. The tensile strength of the sintered nanocomposites primarily depends on the compactness of sintered nanocomposites, homogeneous dispersion of the reinforcement phase, and interface bonding between the reinforcement phase and the matrix (or wettability). With increased content of GNPs, the tensile strength of the sintered nanocomposites exhibited a decreasing trend. There were minor differences in tensile strength between the sintered nanocomposites prepared with 0.2 wt% GNPs or 0.5 wt% GNPs. However, the tensile strength of the sintered nanocomposites decreased by 12% when the content of GNPs increased to 0.8 wt%. This is consistent with the variation trend of the compactness of the sintered nanocomposites. With the increase of the content of GNPs, the agglomeration of the reinforcement phase increases and the extent of heterogeneous distribution in the sintered nanocomposites increases, thus loosening the organization of the sintered nanocomposites [30]. Agglomeration of GNPs and MWCNTs prevents the effects of improved strength and reinforcement, thus decreasing the tensile strength of the sintered nanocomposites. If the extent of agglomeration of the reinforcement phase increases, the extent of irregular microcracks would also increase during the pressing-sintering process. This results in increased stress concentration and decreased effective bearing area of the stress. Finally, the tensile strength of the sintered nanocomposites decreases. Due to the non-wetting between the Cu matrix and GNPs and MWCNTs, the interface bonding is dominated by physical and mechanical bonding. The dispersion is much better and certain interface bonding occurs for low GNPs and MWCNTs. In this study of GNP/MWCNT synergistic action, with an increase of GNPs, the agglomeration extent of the reinforcement phase increased and the interface bonding was weak, thus decreasing the capacity of the reinforcement phase to transmit the load and finally decreasing the tensile strength of the sintered nanocomposites [22].

In summary, co-operative enhancement effects of multi-phase reinforcements were significantly higher than single MWCNT enhancement effects or without GNP/MWCNT synergistic action for the mechanical properties of Cu/Ti_3_SiC_2_/C, Cu/Ti_3_SiC_2_/C/MWCNTs, and Cu/Ti_3_SiC_2_/C nanocomposites reinforced by MWCNTs and graphene. These enhancing effects include grain refinement strengthening, load transfer strengthening, Orowan mechanism strengthening, and large interface strengthening of GNPs.

**Fig. 10:**
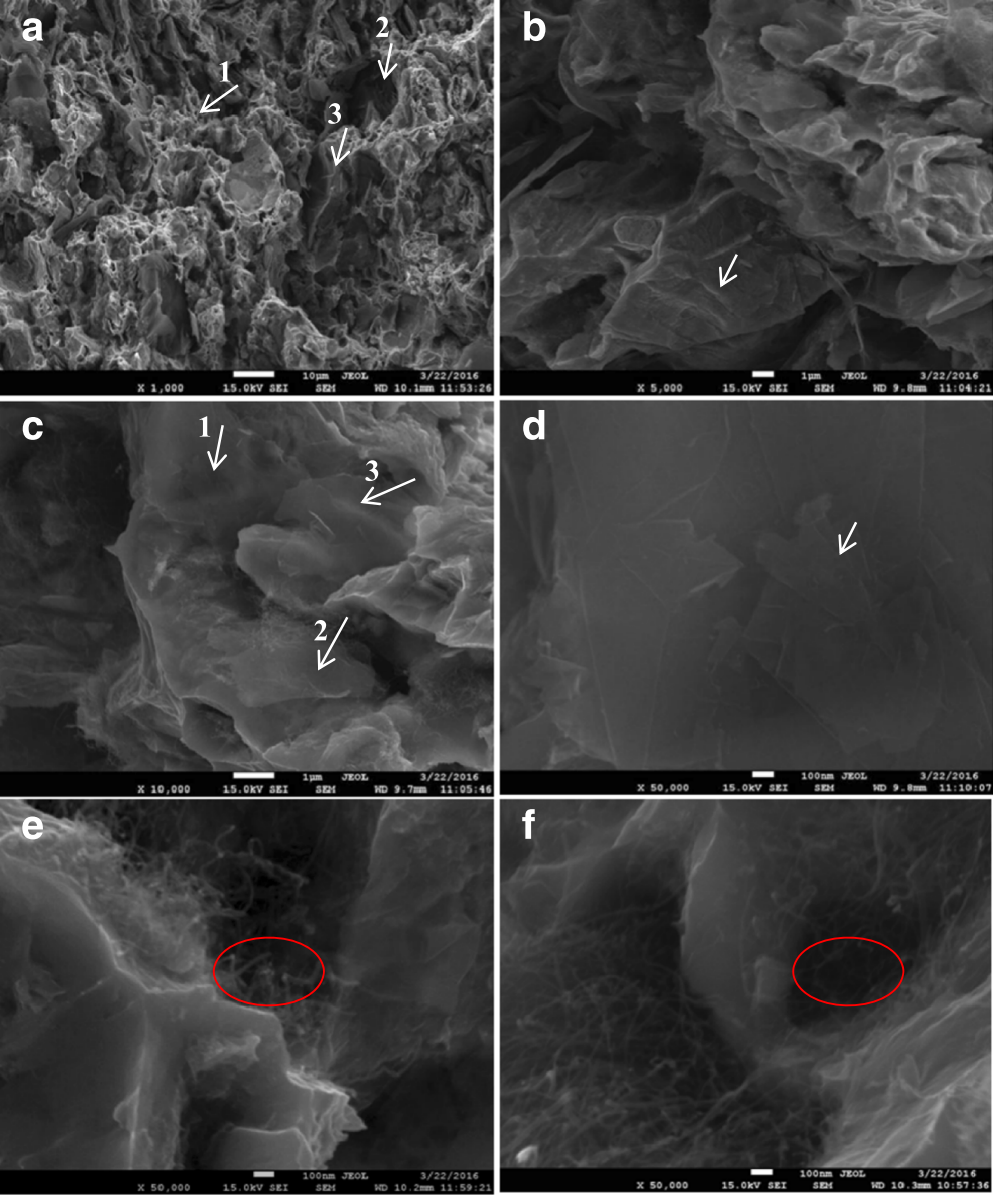
**a**−**f** SEM images of fractures of the sintered nanocomposites with 0.5 wt% graphenes and 0.5 wt% MWCNTs

Figure 10 presents SEM analyses of tensile fracture microscopic process of the nanocomposites. As shown in Figs. 10a, b, the fracture surface of the nanocomposites exhibits a typical dimple and cleavage fracture pattern. Simultaneously, due to non-wetting phenomenon between GNPs, MWCNTs, and the Cu matrix, or due to defects in the sintering process, cracks or holes were evident, as indicated by arrows 2 and 3. This is consistent with the absence of GNPs, MWCNTs, and Cu matrix in the TEM analysis. Stress concentration occurs at cracks or holes of nanocomposites, and microcracks can initiate in these regions to form cracks or holes that can propagate and lead to fracture. Graphene itself has a large specific surface area that increases the contact area with the Cu matrix to promote interface bonding, but also makes it more prone to agglomeration [13]. In Fig. 10c, graphene sheets are pulled out during the tensile test as indicated by arrows 2 and 3. The two arrows show interface bonding between graphene and the Cu matrix. The agglomerated graphene atoms are pulled out from the adjacent micro-cracks at arrow 2, because the agglomeration of graphene leads to the formation of cracks. The graphene is embedded in the Cu matrix because the interface bonding is good between the graphene and the Cu matrix. However, agglomerated graphene sheets can be seen in Fig. 10d and are not effectively transferred during loading in the matrix. Under tensile stress, agglomerations can form micro-cracks and extend sequentially into a crack or form a secondary crack [13, 22, 30]. Additionally, the unique fold structure of graphene can be seen as indicated by the arrow in Fig. 10d. The graphene atoms are first flattened and then rupture when subjected to stress, leading to a certain strengthening-toughening effect. As shown in Fig. 10e, MWCNTs have been embedded in the Cu matrix, explaining MWCNTs good loading transfer ability and the improved tensile strength of the nanocomposites [11]. Nevertheless, as shown in Fig. 10e, f, the disordered aggregation of MWCNTs is observed, and micropores or microcracks are formed in the agglomeration region, which decrease the strength of the nanocomposites.

## Conclusions

The following conclusions can be drawn based on microstructural and mechanical evaluation of Cu/Ti_3_SiC_2_/C nanocomposites reinforced with MWCNTs and graphene.

1. Powder metallurgy techniques (vacuum hot-pressing and hot isostatic pressing) can successfully be applied to synthesize Cu/Ti_3_SiC_2_/C nanocomposites reinforced with MWCNTs and graphene.
2. The synergetic effect of sintered nanocomposites primarily depends on the compactness of the sintered nanocomposites, the homogeneous dispersion of the reinforcement phase, and interface bonding between the reinforcement phase and the matrix.
3. The optimum value of Cu/Ti_3_SiC_2_/C nanocomposites was reinforced with 0.8 wt% MWCNTs and 0.2wt% graphene. When GNPs and CNTs are used as the synergistically reinforced matrix, with the increase of GNPs content, reinforcement agglomeration increasingly affects the strengthening and fracture mechanism of the resulting materials.
4. Enhanced properties of Cu/Ti_3_SiC_2_/C nanocomposites reinforced with MWCNTs and graphene include grain refinement strengthening, load transfer strengthening, Orowan mechanism strengthening, and large interface strengthening of GNPs.

## Abbreviations

EDS: Energy dispersive X-ray spectrometer
HIP: Hot isostatic pressing
MWCNTs: Multi-walled carbon nanotubes
OM: Optical microscopy
SEM: Scanning electron microscope
TEM: Transmission electron microscope
VHP: Vacuum hot-pressing
XRD: X-ray diffraction
